# LexOPS: An R package and user interface for the controlled generation of word stimuli

**DOI:** 10.3758/s13428-020-01389-1

**Published:** 2020-05-11

**Authors:** Jack E. Taylor, Alistair Beith, Sara C. Sereno

**Affiliations:** 1 Institute of Neuroscience and Psychology, University of Glasgow, 62 Hillhead Street, Glasgow, G12 8QB UK; 2 School of Psychology, University of Glasgow, 62 Hillhead Street, Glasgow, G12 8QB UK

**Keywords:** Word stimulus generation and control, Lexicality, Orthography, Phonology, Semantics

## Abstract

LexOPS is an R package and user interface designed to facilitate the generation of word stimuli for use in research. Notably, the tool permits the generation of suitably controlled word lists for any user-specified factorial design and can be adapted for use with any language. It features an intuitive graphical user interface, including the visualization of both the distributions within and relationships among variables of interest. An inbuilt database of English words is also provided, including a range of lexical variables commonly used in psycholinguistic research. This article introduces LexOPS, outlining the features of the package and detailing the sources of the inbuilt dataset. We also report a validation analysis, showing that, in comparison to stimuli of existing studies, stimuli optimized with LexOPS generally demonstrate greater constraint and consistency in variable manipulation and control. Current instructions for installing and using LexOPS are available at https://JackEdTaylor.github.io/LexOPSdocs/.

## Introduction

**Fig. 1:**
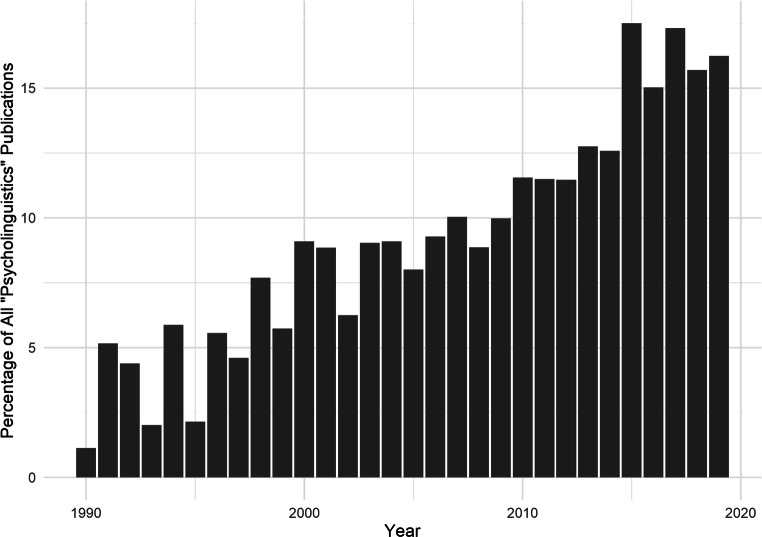
The percentage of documents on Scopus published each year in the period 1990-2019 containing the term “psycholinguistics” in the title, abstract, or keywords, which also contains the term “corpus”, “database”, or “norms”

The number and size of psycholinguistic corpora that have been created and employed in research have greatly increased in recent years. Figure 1 shows the growing proportion of psycholinguistic research over the past three decades that provides or cites databases related to various properties of words. Indeed, the use of large datasets has been made considerably more feasible as a result of the internet and an increase in computing power. Although such large-scale databases of psycholinguistic features, with interfaces for querying and downloading contents, have existed for many years (e.g., Balota et al., 2007; Coltheart, 1981), few tools currently exist to aid in adapting these datasets to generate lexically controlled stimuli, and these are often greatly limited in their capabilities. This makes the generation of suitably controlled word stimuli currently time-consuming and labor-intensive.

This article presents LexOPS, a flexible R package that offers a comprehensive range of capabilities relevant to the generation of psycholinguistically controlled word stimuli. The appellation, ‘LexOPS’, is derived from four types of word properties commonly recognized in psycholinguistics– *Lex*ical, *O*rthographic, *P*honological, and *S*emantic. The most noteworthy feature of LexOPS is that it can produce suitably controlled word stimuli for any possible user-specified factorial design. To support this functionality, the package features an easy-to-use graphical user interface in the form of a Shiny app (Chang, Cheng, Allaire, Xie, & McPherson, 2018), which provides multiple interactive visualizations and summaries of available word properties, as well as how stimuli LexOPS has generated relate to these properties. Another novel feature of the package is that it can work with any database of word variables which the user provides. This means that the user is not limited to built-in variables or words, but can design stimuli according to any numerically or categorically defined properties, for words from any language. Nevertheless, several useful psycholinguistic variables are included from a range of datasets to illustrate the capabilities of LexOPS. These also serve as a template demonstrating the expected format of the data if users wish to run LexOPS on their own databases. Given that LexOPS can work with any suitably formatted data, and the ease with which new datasets can be downloaded and combined, the built-in dataset included with LexOPS is explicitly not exhaustive in its coverage.

This article first provides an overview of the package’s functionality in generating well-controlled stimuli. We then describe the variables native to LexOPS, citing sources for the data and explaining the processes by which original variables were calculated. Using variables drawn from the built-in dataset, we then provide illustrative examples of possible applications for LexOPS. Following this, an introduction to the package’s accompanying Shiny app is presented. We also report the results of a validation analysis, comparing the stimuli used in several well-controlled experiments to examples generated with the package. Implications for reproducibility and replicability are discussed. While we do provide an overview of the package’s functionality, it should be noted that this article is not intended to be read as a tutorial. Detailed instructions on how to install and use the package are available in the LexOPS walkthrough: https://JackEdTaylor.github.io/LexOPSdocs/.

## Functionality overview

LexOPS is designed to support two main methods of stimulus generation: a fully automated grouping of items into factorial cells according to specific constraints (with the “generate pipeline”), and a more bespoke matching of stimuli from several candidates (with the *match_word()* function). Example practical applications are provided with code later in this article.

The “generate pipeline” consists of three main functions: (1) *split_by()*, for specifying independent variables; (2) *control_for()*, for specifying variables that should not differ between conditions; and (3) *generate()*, for running the algorithm that generates lists of stimuli. The factorial designs specified by these functions can adopt any number of word properties, expressed either numerically (e.g., concreteness) or categorically (e.g., part of speech), as independent variables with user-defined levels. Similarly, the user can define any number of control variables, with tolerances of any size. The *generate()* function employs options defined in *split_by()* and *control_for()* to create a stimulus list, with the requested number of items, that fit the specified options.

The *generate()* function creates lists of stimuli in the following way. First, the condition to which items should be matched (i.e., the “match-null” condition), is defined pseudo-randomly, such that each condition is used as a match-null with equal frequency, and in a random order. If the number of stimuli requested is not divisible by the number of conditions, the match-nulls will be allocated as equally as possible across conditions, with over-represented conditions selected randomly. The function then iteratively generates combinations of stimuli. On each iteration, a word is randomly selected that fits the current match-null condition’s specifications (e.g., a word with a low valence rating that is a noun). Possible matches that fit the other conditions’ specifications (e.g., high valence nouns, low and high valence verbs), but that are matched to the word selected from the match-null condition on control variables (e.g., within ± 0.2 Zipf frequency and of equal length), are then identified for each condition, from a pool of unused words. One word is randomly selected from this pool for each condition. If it is not possible to generate a match from each condition for the word from the match-null condition, the function will discard the result of this iteration, and randomly select another word that has not yet been tried, from the same match-null condition. Words that are successfully generated for each condition are stored, and the function will attempt to generate another matched set for the next match-null condition. This will be repeated until as many stimuli are generated as was requested, or until the function fails to generate new stimuli. In addition, the user can elect to generate as many stimuli as possible. If this is specified, the function will generate items until it can no longer generate a matched set across all conditions.

Additional functions and arguments exist to facilitate the generation of stimuli for more complex experimental designs with the *generate()* function. One such function is *split_random()*, which permits the user to generate stimuli with randomly allocated splits. This might be useful for controlling stimuli across stimulus-irrelevant experimental manipulations (e.g., across tasks or contexts). Another such function is *control_for_map()*, a higher-order version of *control_for()* which allows control variables to be calculated within iterations of stimulus generation, relative to the string selected as the match-null. This is useful for controlling similarity or distance values, such as orthographic or phonological Levenshtein distance. Further details and example applications of these functions are available in the online walkthrough. It is also possible to specify different methods of controlling for variables with the *match_null* argument, with words matched relative to one specific condition, relative to a different condition each iteration (selected randomly or pseudo-randomly in equal proportions per condition), or relative to all other conditions. More extensive documentation, highlighting the flexibility of the “generate pipeline”, is available in the online walkthrough.

Finally, LexOPS permits more bespoke stimulus generation, with the *match_word()* function. This function suggests possible matches for a given string, within tolerances for any number of variables specified by the user. This is useful for cases when the automatic stimulus generation detailed above is unsuitable. For instance, experiments presenting stimuli within sentences often require that matched controls for target words are plausible replacements within a given sentential context. The *match_word()* function will return a list of possible matches ordered by Euclidean distance (calculated from all numerical matching variables). The user can then easily select the best match that is a suitable replacement for the target word.

## Inbuilt variables

While the package can generate stimuli from any dataset provided by the user, LexOPS has a dataset already inbuilt. This dataset is not exhaustive, but is an amalgamation of several variables useful for generating word stimuli. These variables can be broadly sorted into five categories: (1) lexical, (2) orthographic, (3) phonological, (4) semantic, and (5) behavioral. Some variables were taken directly from freely available published corpora, whereas others were calculated indirectly from such sources. All built-in variables are for English words only. The package will work with variables from any language, but these need to be provided by the user.

The built-in dataset was filtered, such that word entries were excluded based on the following criteria: (1) they contained non-alphabetic characters; (2) they were longer than 28 characters; or (3) they were only observed once out of all of the word frequency corpora that were used. This left a total of 262,532 unique word strings.

### Lexical variables

Built-in lexical variables include word frequency and part of speech. Word frequency corpora comprise the SUBTLEX-US corpus (Brysbaert & New, 2009), the SUBTLEX-UK corpus (van Heuven, Mandera, Keuleers, & Brysbaert, 2014), and the British National Corpus (BNC; “The British National Corpus, version 3 (BNC XML Edition),” 2007). Frequencies are available in LexOPS in two standardized measures—in frequency per million words (fpmw), or in the Zipf scale, calculated as Zipf = log10(frequency per billion words) (van Heuven et al., 2014). The Zipf scale is a log-normalized measure of word frequency bounded between 1 and 8, which in the context of LexOPS makes it easier to visualize and implement as an independent variable or control variable than fpmw or log(fpmw) (Brysbaert, Mandera, & Keuleers, 2018). The BNC frequencies were calculated by parsing the tagged xml of the latest version of the BNC. LexOPS additionally separates the written and spoken sources in the BNC, though the combined frequency across these modalities is also available.

The part of speech for a given word in LexOPS is defined as its most commonly identified part of speech within a specific corpus. Part of speech is available as a categorical variable, according to SUBTLEX-UK, the BNC, and the English Lexicon Project (ELP; Balota et al., 2007).

### Orthographic variables

Inbuilt orthographic variables consist of length (number of characters), bigram probability, and orthographic neighborhood size.

Character bigram probability was calculated using the word frequency corpora listed in the previous section. For each word frequency corpus, the probability of each possible character bigram (from *aa* to *zz*) was calculated by counting the number of times each bigram appears, weighted by the frequency of the words it appeared in, in fpmw. These bigram frequencies were then scaled from 0 to 1 to get the respective probabilities of all bigrams. A word’s bigram probability could then be calculated as the mean probability of all its constituent bigrams (i.e., both overlapping and non-overlapping).

Orthographic neighborhood size is available in two measures. The first is Coltheart’s *N* (Coltheart, Davelaar, Jonasson, & Besner, 1977), defined as the number of words at a Hamming distance of 1 (i.e., a one-character substitution) from a given word.. The second is Orthographic Levenshtein Distance 20 (OLD20; Yarkoni, Balota, & Yap, 2008), defined as the mean Levenshtein distance between a given string and its 20 closest Levenshtein neighbors, where Levenshtein distance is the minimum number of character insertions, substitutions, or deletions between two strings. The OLD20 measure is generally preferable to Coltheart’s *N*, as it allows for distance calculation between strings of different lengths. Both of these measures were calculated using the R package, “vwr” (i.e., “visual word recognition”; Keuleers, 2013).

### Phonological variables

The inbuilt phonological variables of LexOPS comprise the following: number of phonemes, number of syllables, number of pronunciations, rhyme, and phonological neighborhood size. The phonological features were calculated using phonetic transcriptions from two different sources: the eSpeak speech synthesizer’s (“eSpeak version 1.48.15,” 2015) standard British English pronunciations of all entries in the database; and the Carnegie Mellon University (CMU) Pronouncing Dictionary of American English (Weide, 2014).

The transcription system adopted by eSpeak (Kirshenbaum phonetic encoding) uses one-character ASCII representations for individual phonemes, but two-character representations for affricates and diphthongs. The affricates /ʧ/ and /ʤ/ (as in the beginnings of *char* and *jar*, respectively) are encoded with one-character ASCII representations. The CMU transcriptions are represented by an ARPAbet transcription system for American English, and are represented as either two-letter or one-letter ASCII characters. For example, the word *how*, containing the diphthong /aʊ/, is represented as ‘HOW’ in the two-character system or as ‘hW’ in the one-character system. Similarly, the word *China*, containing the affricate /ʧ/, is represented as ‘CH-AY-N-AE’ (with phonemes separated by hyphens) in the two-character system, or as ‘CYN@’ in the one-character system.

Number of phonemes is simply a count of how many phonemes a word contains. The number of syllables was calculated by simply counting the number of vowel phonemes that occurred in the transcription. The number of pronunciations is a variable only available for the CMU Pronouncing Dictionary, calculated by simply counting how many possible pronunciations are listed for each entry. This includes differences in both pronunciation and stress patterns.

Rhyme is represented as a categorical variable consisting of a transcription of all phonemes from the final vowel phoneme until the end of the word (i.e., the final syllable’s ‘rime’). For instance, eSpeak’s British English pronunciation of *partake* is represented as /pɑteɪk/ in the International Phonetic Alphabet (IPA) and, as such, belongs to the rhyme category of /-eɪk/, which it shares with entries such as *steak* and *opaque*.

Phonological neighborhood size is available in terms of the phonological Coltheart’s *N* and Phonological Levenshtein Distance 20 (PLD20), calculated similarly to the orthographic neighborhood measures, using the “vwr” package for R (Keuleers, 2013).

### Semantic variables

Semantic features which LexOPS has built-in mostly come from norming studies in which participants provide ratings for a particular semantic aspect of a word on a Likert scale. A summary of the available semantic features is presented in Table 1.

**Table 1:** Summary of the sources and semantic features used in LexOPS

Source and semantic features	Scale	*N* words	Observations/Word^a^
Scott, Keitel, Becirspahic, Yao, & Sereno (2019)			
AROU	1–9	5553	33.31 (3.72)
VAL	1–9	5553	33.54 (3.73)
DOM	1–9	5553	33.24 (3.73)
CNC	1–7	5553	33.34 (3.80)
IMAG	1–7	5553	33.30 (3.74)
FAM	1–7	5553	32.36 (3.60)
AOA	1–7	5553	33.94 (3.69)
SIZE	1–7	5553	33.30 (3.79)
GEND	1–7	5553	33.25 (3.85)
Warriner, Kuperman, and Brysbaert (2013)			
AROU	1–9	13,915	22.97 (23.73)
VAL	1–9	13,915	21.81 (23.44)
DOM	1–9	13,915	24.32 (25.07)
Brysbaert, Warriner, and Kuperman (2014)			
CNC	1–5	37,058	at least 25
Clark and Paivio (2004)			
IMAG	1–7	2311	47–49
FAM	1–7	2311	16
Kuperman, Stadthagen-Gonzalez, and Brysbaert (2012)			
AOA	ages 1–25	30,124	18–22 for most items
Brysbaert and Biemiller (2017)			
AOA^b^	ages 2–14^c^	43,991	around 200
Engelthaler and Hills (2018)			
HUM	1–5	4997	32.93 (5.64)

For each source, the relevant semantic feature(s), scale, number of words, and observations per word are specified

*AROU* arousal; *VAL* valence; *DOM* dominance; *CNC* concreteness; *IMAG* imageability; *FAM* familiarity; *AOA* age of acquisition; *SIZE* semantic size; *GEND* gender association; *HUM* humor

^a^Where the number of observations for each word was available, the mean, and standard deviation in parentheses, are presented; otherwise, summary statistics are reported. ^b^This measure is test-based, not from a rating study. ^c^Age estimates cover ages 2, 4, 6, 8, 10, 12, 13, and 14

### Behavioral variables

Behavioral variables consist primarily of lexical decision response time and accuracy from the ELP (Balota et al., 2007) and the British Lexicon Project (BLP; Keuleers, Lacey, Rastle, & Brysbaert, 2012).

Behavioral variables also include measures of proportion known (the proportion of people who know a given word) and word prevalence (probit-transformed proportion known), taken from Brysbaert, Mandera, and Keuleers (2019). Brysbaert et al. (2019) demonstrate that proportion known and word prevalence have advantages over variables such as word frequency, age of acquisition, and familiarity (which have traditionally served as proxies to gauging word difficulty) since these two measures more directly operationalize word difficulty.

## Example applications

As an example, a user could define a 2 × 2 design to investigate the interaction between character bigram probability, according to SUBTLEX-UK, and concreteness ratings, according to Brysbaert et al. (2014). The user could also specify that stimuli should be controlled across conditions for word frequency within ±0.2 Zipf according to SUBTLEX-UK, as well as exact word length. The dataset that stimuli are generated from can be additionally filtered, for instance according to word prevalence reported by Brysbaert et al. (2019) such that the generated stimuli consist entirely of words that at least 90% of people know. The following R code will generate 50 words per factorial cell (200 in total) that fit these specifications. The variables used in this example have all been drawn from the inbuilt dataset described in the previous section to make the code more easily readable.

stim <- lexops %>%

**subset**(PK.Brysbaert >= 0.9) %>%

**split_by**(BG.SUBTLEX_UK, 0:0.003 ~ 0.009:0.013) %>%

**split_by**(CNC.Brysbaert, 1:2 ~ 4:5) %>%

**control_for**(Length, 0:0) %>%

**control_for**(Zipf.SUBTLEX_UK, -0.2:0.2) %>%

**generate**(n = 50)

**Fig. 2:**
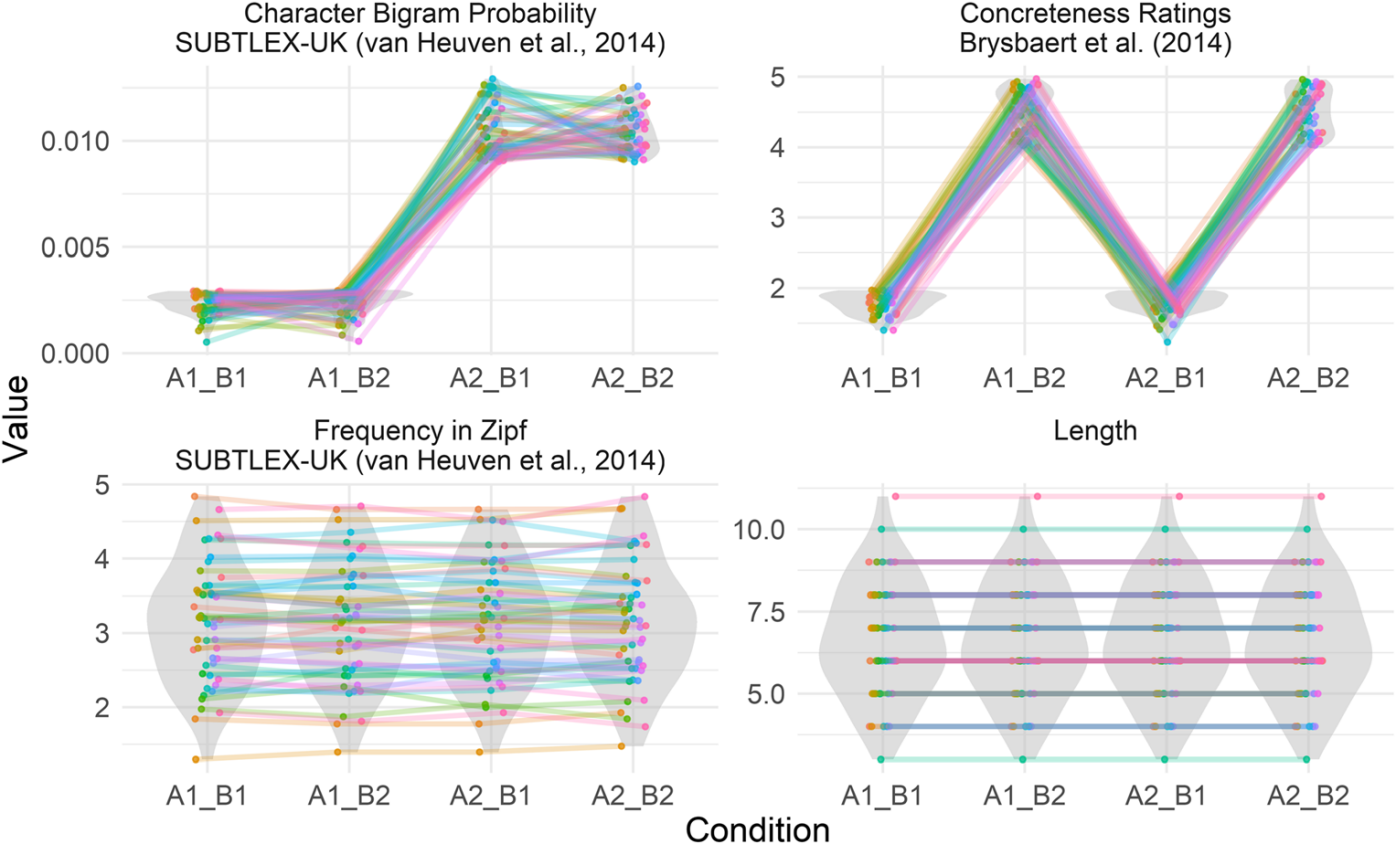
An example figure generated by the *plot_design()* function, for a stimulus list generated by the example code, consisting of 200 words split into four factorial cells: A1_B1 (low bigram probability, low concreteness), A1_B2 (low bigram probability, high concreteness), A2_B1 (high bigram probability, low concreteness), and A2_B2 (high bigram probability, high concreteness). In this example, words are controlled in terms of frequency (within ±0.2 Zipf), and length (exactly). When words are more closely matched on a variable, the distributions of control variables appear more similar, and the slopes of lines between matched items are less steep. The differences between conditions in character bigram probability and concreteness ratings (sought by the user) are reflected in the upper two plots

The distributions of generated stimuli on relevant numerical variables can be readily examined using the *plot_design()* function. Figure 2 presents an example figure generated by the *plot_design()* function for a stimulus list generated by the code above. This function produces a multi-faceted figure showing the distributions (in violin plots) of all numeric independent or control variables used for each generated condition. Within each distribution, individual words are visualized as points, joined by lines to other words (points) from the same matched set (i.e., that share the same match-null). The figure can be a quick way to check that LexOPS has generated stimuli as expected. For instance, excessive differences between generated conditions in the distributions of control variables may indicate that more restrictive tolerances might be appropriate.

The *match_word()* function is convenient in cases where matches need to be controlled for factors that would be difficult to include as numeric or categorical variables, such as maintaining sentence plausibility when a target word is replaced. As a practical example, imagine an experiment where the researcher wants to replace target words in existing sentences with words having a later age of acquisition. Suppose they also want the words to be controlled for length, frequency, concreteness and part of speech (according to the written texts of the BNC). If the researcher wanted to find a suitable replacement for the word “butterfly” in the sentence, “The man had never seen such an enormous butterfly before”, they could use the following code to identify a suitable match. Again, all the variables used have been drawn from the inbuilt dataset for readability.

suggested_matches <- lexops %>%

**match_word**(

"butterfly", Length,

Zipf.SUBTLEX_UK = -0.2:0.2,

CNC.Brysbaert = -0.25:0.25,

PoS.BNC.Written

) %>%

**subset**(AoA.Kuperman >= 9)

This would return a data frame containing four possible matches, ordered by Euclidean distance in the matching variables: “satellite”, “orchestra”, “champagne”, and “machinery”. Of these, the researcher would probably select the word “satellite”, as the closest match that is a plausible replacement for “butterfly” in the sentence.

## The Shiny app

**Fig. 3:**
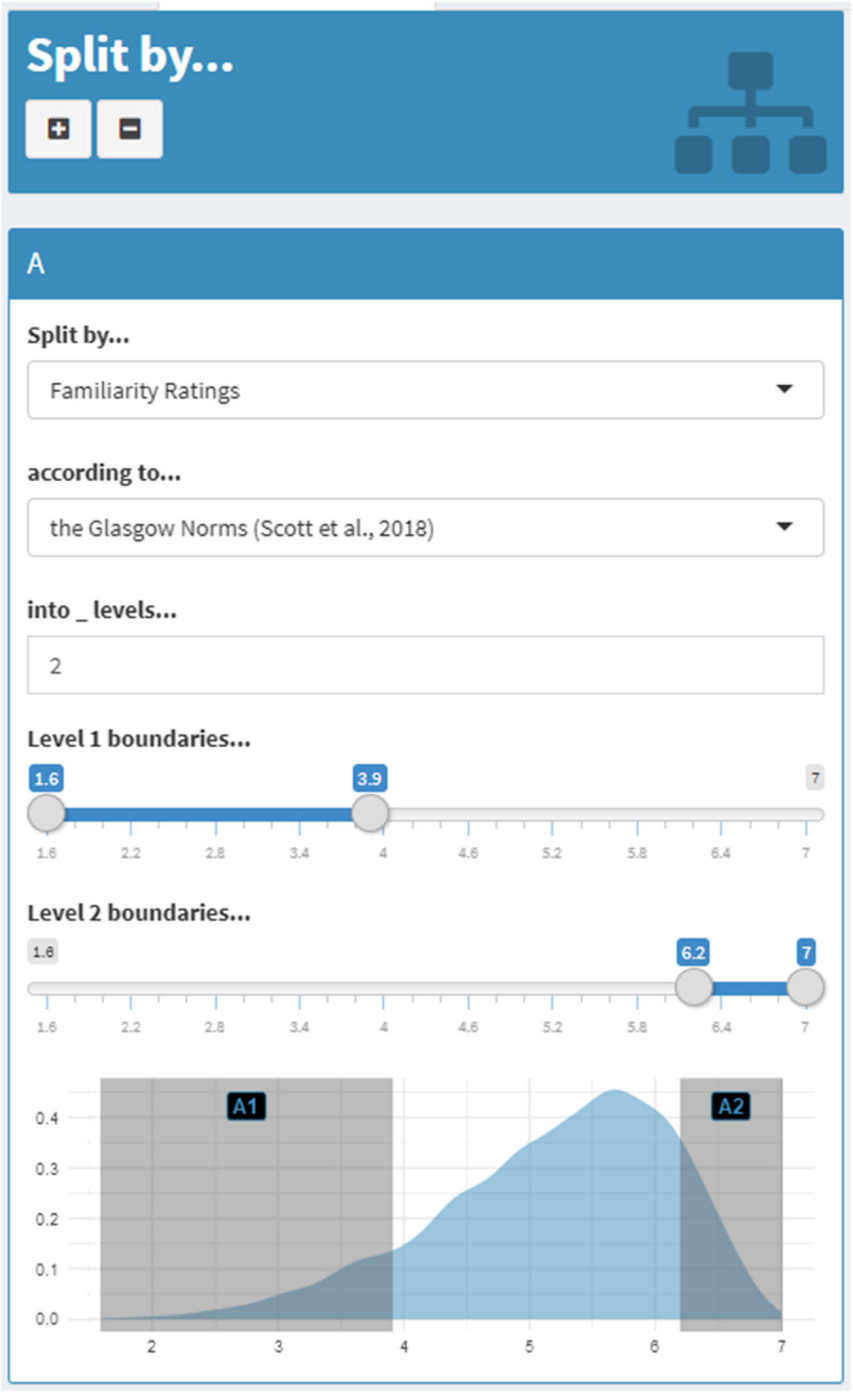
An example box for specifying the levels of an independent variable in the Shiny app. Here, two levels (A1, A2) are being specified for the variable of Familiarity from the Glasgow Norms (Scott et al., 2019). In this case, the density plot shows that the distribution is skewed towards words rated as more familiar, with far fewer words rated as less familiar. As such, it might make sense to use a wider range or bin for a low familiarity condition, to ensure there are enough candidate words. Similar boxes are used for specifying controls and filters. Such boxes can be added to or removed from the design specification with the plus and minus buttons, respectively

LexOPS features a graphical user interface in the form of a Shiny app (Chang et al., 2018), which provides an interactive front-end to the package’s functions. For instance, tolerances for independent variables can be specified via a slider (i.e., a moveable graphical button on an analogue scale), and are then visualized as shaded areas in a plot of a variable’s density. Figure 3 presents such an example for defining experimental conditions in the *split_by()* function. The “generate pipeline” is accessible through a “Generate” tab in the sidebar, while the *match_word()* function is accessible through a “Match Word” tab. Interactive functionality is also provided for querying the LexOPS dataset (through the “Fetch” tab), and for integrating custom variables or datasets into the app (through the “Custom Variables” tab). The Shiny app’s graphical user interface is likely to be more accessible for users unfamiliar with R, as it can be run with a minimal amount of R code with the *run_shiny()* function, though we believe that the speed and ease with which it allows for stimulus generation make it a convenient feature for all users. Furthermore, the Shiny app automatically translates the user’s selections into reproducible R code that can then be run as a stand-alone R script.

In addition to providing an interface to LexOPS functions, the Shiny app also provides an interface in its “Visualise” tab for interactive visualization of relationships between variables, and the distribution of generated stimuli across variables. Here, users can select variables to plot on *x*- and *y*- axes, and can optionally elect to plot variables on a *z*-axis or color scale. LexOPS will generate an interactive scatter plot of all words which have a value for all requested variables, where each point represents a single word. By hovering with the cursor over a given point, the user can query the word visualized at that location as well as its specific values (coordinates) across the plotted variables.

**Fig. 4:**
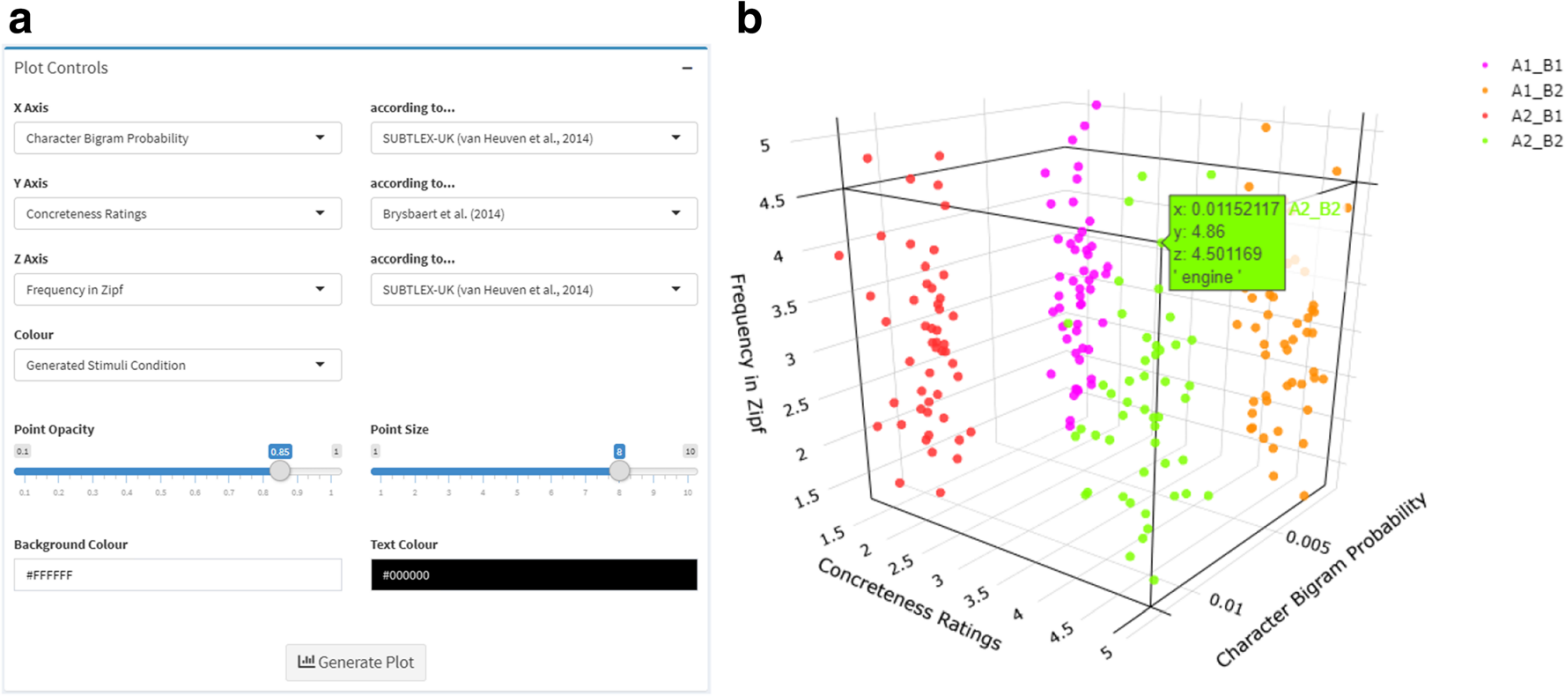
Example showing **a** user interface options and **b** resulting interactive plot produced by the Visualise tab, for stimuli generated by the “generate pipeline” specified by the code in the Example Applications section (2 × 2, character bigram probability by concreteness design, controlling for length and frequency). Each *point* corresponds to one word, which can be queried by the user by moving the cursor directly over that point. In the example, the user has queried a word from condition A2_B2, corresponding to the word, “engine”

Whereas axes can only be used to visualize numerical values, color scales can be used to visualize the distributions of variables which are either numerical or categorical. For instance, the user can select to view the distributions of different parts of speech by means of differential coloring of the defined levels of this variable. The user can also have the app visualize distributions of stimuli produced by the Generate tab, as shown in Fig. 4, as well as suggested matches produced by the Match tab, or words uploaded to the Fetch tab.

## Validation

To demonstrate that the package is a valuable tool for generating word stimuli, we tested whether LexOPS could produce stimulus sets comparable to those of previous studies that employed well-controlled word stimuli. Four studies were selected based on the following criteria: the experimental design was unambiguously presented (e.g., with clear definitions and/or boundaries of conditions); the characteristics of stimuli (e.g., concreteness, valence) were taken from freely available published norms; the stimuli across conditions were matched on an item-by-item basis; and the complete set of stimuli was provided. The first study, by Kousta, Vigliocco, Vinson, Andrews, and Del Campo (2011), examined concreteness (high/low), using 38 words per condition, and controlling for 12 different psycholinguistic variables. The second study, by Scott, O’Donnell, Leuthold, and Sereno (2009), investigated the interaction between word frequency (high/low) and emotional valence (negative/neutral/positive), using 40 words per each of the six conditions, and controlling for word length and frequency. The third study, by Sereno, Scott, Yao, Thaden, and O’Donnell (2015), employed a similar frequency (high/low) by emotion (negative/neutral/positive) design, with a different set of 40 words per condition, and similarly controlled for word length and frequency. Finally, Yao et al. (2018) examined the interaction between concreteness (high/low) and emotion (negative/neutral/positive), using 45 words per factorial cell, and controlling for word length and frequency.

**Fig. 5:**
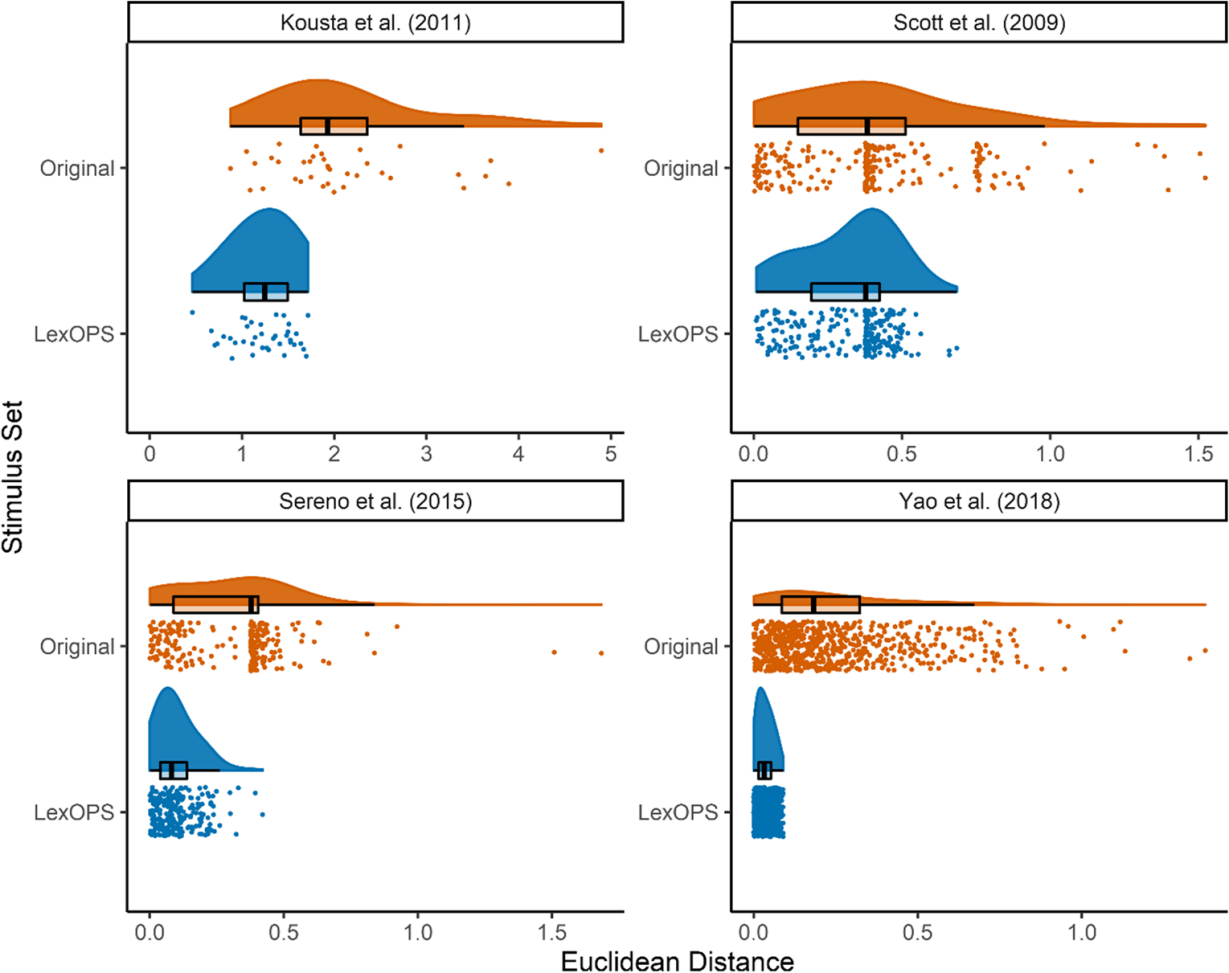
The Euclidean distance values between each matched pair of words in the four studies, for the original study (in *orange*) and the stimuli generated by LexOPS (in *blue*). Each *point* represents a single value of distance, while the density plot above depicts the shape of the distribution. The overlaid *boxplots* present summary statistics of the median (*central, dark vertical line*), first and third quartiles (*the left- and right-most ends of the boxes*) and the range of the values, bounded to within a distance of 1.5 times the interquartile range from the boxes (*the whiskers*). The bands of points seen in the values for Scott et al. and Sereno et al. reflect that stimuli were allowed to differ in length. The bands are absent in the distance values for the LexOPS stimuli generated for Sereno et al., as these stimuli were matched for length exactly

For each study, we used LexOPS to generate the same number of stimuli according to the original constraints that had been specified. We used the same databases that were detailed within the studies with one exception (the norms for one of Kousta et al.’s control variables, context availability, were obtained locally and were not freely available). In all cases, LexOPS was able to generate stimuli that fit within the boundaries of the original conditions, which were matched at least as closely on all control variables. In many cases, it was found that closer tolerances on many variables were possible than those implemented in the original studies. To encapsulate the comparison between the original stimuli and those generated by LexOPS, for both lists the Euclidean distance in all numeric control variables (scaled by standard deviation for comparability) was calculated between each word in the list, and each word it should be matched to. As the controls were implemented item-wise, this resulted in $n\frac{k\left(k-1\right)}{2}$ observations of Euclidean distance for each stimulus list, where *n* is the number of items per factorial cell, and *k* is the number of factorial cells. The calculated values are presented in Fig. 5.

## Reproducibility and replicability

LexOPS offers a valuable contribution to research in terms of reproducibility and replicability. By sharing LexOPS code, for example in existing repositories such as the Open Science Framework and GitHub, researchers can provide the exact specifications, in readable code, used to generate stimuli lists that were found to produce a given effect. Moreover, the code can include a random seed that allows other users to reproduce a specific stimulus list. If a random seed is not set, or is set to a different value, a given pipeline will generate a different set of stimuli each time it is run. This means that an experimental design can be replicated, with the same relationships between variables but consisting of different stimuli. Other users can also modify shared code to see how such changes in the experimental design might alter a reported effect, for instance, by modifying the cut-off values of a variable’s levels or the tolerances of control variables, or by including additional control variables.

## Applications to other areas of research

Although LexOPS was developed for experiments employing word stimuli, the package can also be used to generate stimuli in any experimental domain for which there is a finite set of possible stimuli, having properties that have been coded numerically or categorically. For example, the Chicago face database (Ma, Correll, & Wittenbrink, 2015) is a resource that specifies both objective and subjective measures of a set of faces. LexOPS could be used on this database to generate stimuli to investigate, for example, a possible effect of attractiveness on face recognition processes. Analogous to its functionality with words, LexOPS could easily be adapted to define levels of facial attractiveness, while controlling for variables such as the race, gender, and luminance of individual faces.

## Discussion

We believe that LexOPS is a valuable resource to researchers who use word stimuli, providing a method for flexible and controlled generation of items, with the added value of intuitive interfaces. In addition, LexOPS facilitates the reproducibility and replicability of experiments, allowing specific stimulus lists to be recreated, and providing an easy method for generating novel stimulus lists for the purposes of replication.

One point that should not be overlooked is that LexOPS is still limited by the nature of the variables, tolerances, and condition boundaries that are used. For instance, some variables have entries for relatively few words (e.g., Clark and Paivio's (2004) norms provide familiarity ratings for only 2311 words), and there is often limited overlap of items between different corpora. This means that if variables from small corpora, or from multiple corpora with little overlap, are used as independent or control variables, the pool of possible stimuli will be greatly reduced. Similarly, variables are often highly correlated, for instance, as imageability and concreteness are (Scott et al., 2019). It would be difficult to generate stimuli for designs probing interactions between such highly correlated variables, or for those in which independent variables and control variables are highly correlated. Finally, the precision of control variables’ tolerances, and the positioning of independent variables’ boundaries relative to the variables’ density distributions, will also modulate the number of possible stimuli that can be generated.

While the features detailed here are unlikely to change, work will continue on the package, and it is very likely that we will add extra functionality to LexOPS in the future in response to users’ requests. Similarly, we may expand the inbuilt database to include further variables if they are likely to be of use to many researchers. Any such additions or changes will be described in the package’s documentation and in the LexOPS walkthrough.

To conclude, we have developed and made freely available a flexible and intuitive tool for the controlled generation of word stimuli. This R package allows researchers to robustly generate lists of stimuli for factorial designs in a reproducible and replicable manner. We expect LexOPS to be of great benefit to a broad range of researchers, particularly those who use word stimuli.
